# Rocks, lichens, and woody litter influenced the soil invertebrate density in upland tundra heath

**DOI:** 10.1371/journal.pone.0282068

**Published:** 2023-05-02

**Authors:** Olukayode O. Jegede, Katherine M. Standen, Steven Siciliano, Eric G. Lamb, Katherine J. Stewart

**Affiliations:** 1 Department of Soil Science, University of Saskatchewan, Saskatoon, Canada; 2 Department of Plant Science, University of Saskatchewan, Saskatoon, Canada; Feroze Gandhi Degree College, INDIA

## Abstract

Soil invertebrates are an integral part of Arctic ecosystems through their roles in the breakdown of litter, soil formation, and nutrient cycling. However, studies examining soil invertebrates in the Arctic are limited and our understanding of the abiotic and biotic drivers of these invertebrate communities remains understudied. We examined differences in soil invertebrate taxa (mites, collembolans, enchytraeids) among several undisturbed upland tundra heath sites in Nunavut Canada and identified the drivers (vegetation and substrate cover, soil nutrients and pH) of the soil invertebrate community across these sites. Soil invertebrate densities were similar to that of other Arctic studies. While invertebrate communities were relatively consistent between our sites, cover of rocks, woody litter, and the lichen *Alectoria nigricans* had significant, positive influences on the density of all invertebrates studied. Mites and collembolans were more closely associated with cover of lichens, whereas enchytraeids were more closely associated with woody litter and rocks. Our results suggest that anthropogenic (e.g., resource exploration and extraction) and/or natural (e.g., climate change) disturbances that result in changes to the vegetation community and woody litter inputs will likely impact soil invertebrates and the ecosystem services they provide.

## Introduction

The Arctic tundra is characterized as a very cold biome with low precipitation, short growing seasons of approximately 50 to 60 days, and poor soil nutrient conditions [1]. Due to these harsh conditions, biodiversity in the Arctic tundra tends to be lower than other biomes [2, 3]. Among those organisms able to persist in such conditions, soil invertebrates make up a substantial proportion [2]. The existence and activities of these soil invertebrates on the tundra are influenced by their interactions with biotic (e.g., vegetation) and abiotic factors (e.g., soil physicochemical conditions; Høye and Culler [4], and they tend to be sensitive to environmental change [4, 5]. As such, disturbance in this region may lead to changes in soil invertebrate communities, which in turn could drive changes in ecosystem services.

Soil invertebrates are integral parts of ecosystems as they are involved in the breakdown of litter, soil formation, and cycling of nutrients [6]. Their activities are crucial to many ecosystem services such as organic matter decomposition, water filtration, and carbon sequestration [5]. In the Arctic tundra, soil micro- and mesofauna (e.g., microarthropods and enchytraeids) are the prominent invertebrates involved in soil formation and decomposition processes [3]. These soil invertebrates also play critical roles in Arctic food webs as prey for other predatory macroinvertebrates like beetles and spiders [3, 7]. However, soil invertebrate data are sparse in low Arctic tundra [8–11]; thus, it is critical to characterize soil invertebrate communities to provide a baseline for understanding potential changes in ecosystem services with natural (e.g., climate change) and anthropogenic (e.g., resource exploration and extraction) disturbances.

Soil invertebrates are influenced by various environmental characteristics, including ground cover (both vegetation and non-living cover like plant litter, rocks, and soil) and soil characteristics like nutrients and pH [12, 13]. For example, soil nutrients and pH are positively correlated with soil invertebrate diversity [13]. Similarly, Sinsabaugh *et al*. [14] reported that soil pH influences enzyme activities which in turn drives soil organism diversity. In addition, vegetation cover influences soil invertebrates: Salman and Blaustein [15] found that the abundance and richness of arthropods increased with structural complexity of vegetation community. Importantly, soil invertebrates can be highly sensitive in their responses to environmental changes [4]. As such, understanding the connectivity between soil fauna, flora, and soil nutrient conditions of the understudied Arctic tundra is important for determining the influence of disturbance on the critical ecosystem services provided by soil fauna.

To address these knowledge gaps, our exploratory study: i) examines differences in soil invertebrate taxa (mites, collembolans, enchytraeids) among several undisturbed upland tundra heath sites and ii) identifies the drivers (vegetation and substrate cover, soil nutrients and pH) of soil invertebrate community across undisturbed upland tundra heath sites. Combined, this information provides key baseline data to better understand the effects of disturbance across upland tundra heath sites on soil invertebrates and the ecosystem services they provide.

## Methods

### Site description

Our study was conducted at the Agnico Eagle Mines (AEM) Meliadine site (63°01’22.9"N, 92°11’41.1"W) within the Kivalliq region of Nunavut Territory, Canada, roughly 25 km from Rankin Inlet. The mean annual precipitation and air temperature of the region are 316 mm and -10.5°C, respectively [16]. Vascular vegetation across the region includes a mix of tundra shrubs (e.g., *Salix* spp., *Cassiope tetragona*, *Dryas octopetala*, *Arctostaphylos alpina*, *Rhododendron tomentosum*) and forbs (e.g., *Chamaenerion latifolium*, *Saxifraga oppositfolia*, *Astragalus alpinus*, *Pedicularis flammea*). Common nonvascular species include lichens (e.g., *Cetraria* spp., *Thamnolia vermicularix*) and numerous bryophytes (e.g., *Hylocomium splendens*, *Aulacomnium spp*., *Racomitrium lanuginosum*, *Pohlia* spp., *Ceratodon purpureus*, *Bryum* spp., and *Distichium* spp.) [17, 18]. Soils are characterized as cryosolic and topped with thick organic layers. Within this region, we selected four study sites (EMP = Site A, Q1 = Site B, Q2 = Site C, Monolith = Side D) in undisturbed tundra heath (S1 Fig).

### Field sampling

At each of the four upland tundra heath sites (Table 1), we randomly selected 8–10 locations for vegetation, soil, and invertebrate sampling. Sampling locations represented local vegetation and had an organic layer depth of at least 10 cm. In July 2019, we determined percent cover of all plant species via visual estimation using a 0.16 m^2^ gridded quadrat (25 grids, 0.0064 m^2^ each) at each sampling location in Sites A, B, and C, whereas at Site D, we used a 0.096 m^2^ gridded quadrat (15 grids, 0.0064 m^2^ each). Sampling quadrat size differed between the study sites due to differences in the available instruments at the time of sampling. Sites A, B, and C were sampled in conjunction with another study occurring at these sites, whereas Site D was added to bolster our dataset and the same quadrat was not available for sampling. Values ranging from 0 to 4 (0 = 0%, 1 = 1–25%, 2 = 25–49%, 3 = 50–75%, 4 = 75–100%) were used for estimation of vegetation and substrate (i.e., litter, rocks, bare soil, etc.) cover in each grid. To ensure data from differently sized quadrats were comparable, the values were subsequently summed and converted to percent cover for the entire quadrat. subsequently summed and converted to percent cover for the entire quadrat. All vascular and non-vascular plants were identified to genus-level and, where possible, to species-level. Soil samples were collected at each location by taking 5 cm diameter and 10–20 cm depth cores containing organic and mineral soil.

**Table 1:** Summary statistics of invertebrate (enchytraeids, collembolans, and mites) densities (m^-2^) across four upland tundra heath sites near Rankin Inlet, NU, Canada, as well as across all sites. Presented are the mean ± standard deviation and, in brackets, the minimum and maximum values.

Site	Enchytraeids	Collembolans	Mites
**A**	44 509 ± 51 444 (0, 161 574)	49 573 ± 69 102 (0, 241 118)	36 300 ± 15 521 (15 310, 54 918)
**B**	29 890 ± 22 796 (666, 69 896)	25 380 ± 19 483 (6657, 71 560)	41 595 ± 26 979 (6657, 80 051)
**C**	40 187 ± 45 445 (0, 145 549)	33 640 ± 23 908 (5713, 90 057)	9994 ± 9502 (714, 29 397)
**D**	27 007 ± 21 628 (5211, 66 692)	20 611 ± 13 211 (7214, 36 133)	41 075 ± 12598 (24 930, 66 841)
**All Sites**	35 840 ± 37 588 (0, 161 574)	32 916 ± 39 359 (0, 241 118)	31 653 ± 21 722 (714, 80 051)

### Invertebrate extraction

Invertebrates were extracted from all soil cores using the water flotation method [19]. To increase the efficiency of invertebrate extraction, we used LUDOX® AM colloidal silica 30% wt. suspension in water. Invertebrates were viewed under a stereomicroscope, counted, and, where possible, identified immediately. Remaining invertebrates were stored in 70% ethanol for subsequent identification. The abundance of invertebrates as mites, collembolans, and enchytraeids collected from the study was determined as density (# of invertebrates in m^-2^).

### Soil chemical analysis

Mineral soil layers from each core were air-dried for determination of nutrient concentrations and pH. To measure pH, we placed 5 g of soil in a 50 ml centrifuge tube with 25 ml of 0.01 M CaCl_2_ and shook the mixture for 30 minutes. We let the mixture settle for two hours before using a Mettler Toledo Orion Dual Star to determine pH [20]. Soil cation and anions were determined by the method described by Quevauviller [21]. For this method, 2.5 g of soil into a centrifuge tube with 25 mL Milli-Q water and shaken for 3 hours at 15 rpm using a rotary shaker. Samples were then centrifuged at 5000 g for 10 minutes and subsequently filtered with a 0.45 μm filter. Afterwards, samples were analyzed for anions (Dionex IonPac AS18 column) and cations (Dionex IonPac CS12A column) with ion chromatography with a DionexICS-2000 using Chromeleon7 software in the Department of Soil Science of the University of Saskatchewan, Saskatoon, Saskatchewan, Canada.

### Statistical analysis

All analyses were conducted in *R* v. 4.1.2 [22] and figures were created using *ggplot2* [23] and *ggpubr* [24]. To compare individual invertebrate density as well as total invertebrate density among the four study sites, we used one-way analysis of variance (ANOVA) and, when significant, Tukey’s Honestly Significant Difference test for pairwise comparisons. Model residuals were visually inspected using *DHARMa* [25]. Data were square root transformed when necessary (i.e., mite and collembolan density) to meet ANOVA assumptions. One location from Site A was removed from further analysis due to an extreme outlier in collembolan density.

Before analysis, we applied the Hellinger transformation to all vegetation and substrate cover data. Afterwards, we conducted principal components analyses (PCA) using *vegan* [26] to visualize differences in vegetation cover, soil nutrients and pH, and substrate cover among the four study sites. Using the *adonis* function (package: *vegan*), we conducted permutational analyses of variance (PERMANOVAs) to assess statistical differences in multivariate data among sites. To determine whether significant PERMANOVA results were due to differences among centroids and/or differences in variance around the centroids (i.e., beta dispersion) for the sites, we used the *betadisper* function (package: *vegan*).

To model the effects of vegetation cover on invertebrate community across our study studies with a redundancy analysis (RDA), we first ensured there was adequate representation of each vegetation species within each site and a large enough range of these species among sites. As such, we removed vegetation species that: 1. had a low range of percent cover (<10%) and/or 2. were absent from 2 or more sites. Because of the vast number of variables that remained, we conducted correlation matrices on each category of variable to further remove highly correlated variables (r > 0.6, r < -0.6). All substrate covers, four of 10 soil nutrient variables (i.e., pH, NH_4_^-^, SO_4_^-^, PO_4_^-^), and 15 of 52 vegetation species remained (see S1 Table). To determine key drivers of invertebrate community among our study sites, we conducted a partial RDA with “site” as a conditioning factor. All remaining variables were scaled. Due to the exploratory nature of our study, we used function *ordistep* (package: *vegan*) to conduct stepwise selection of all remaining soil nutrient, substrate cover, and vegetation cover variables. After variable selection, we ran an RDA in *vegan* and used variance inflation factor (VIF) to assess multicollinearity of explanatory variables. VIF < 3 was considered acceptable. To determine significance (p < 0.05) of each explanatory variable and each axis in the model, as well as significance and goodness-of-fit of the entire model, we used *permutest* and *RsquareAdj* (package: *vegan*), respectively.

## Results

### Invertebrate taxa density among sites

**Fig 1:**
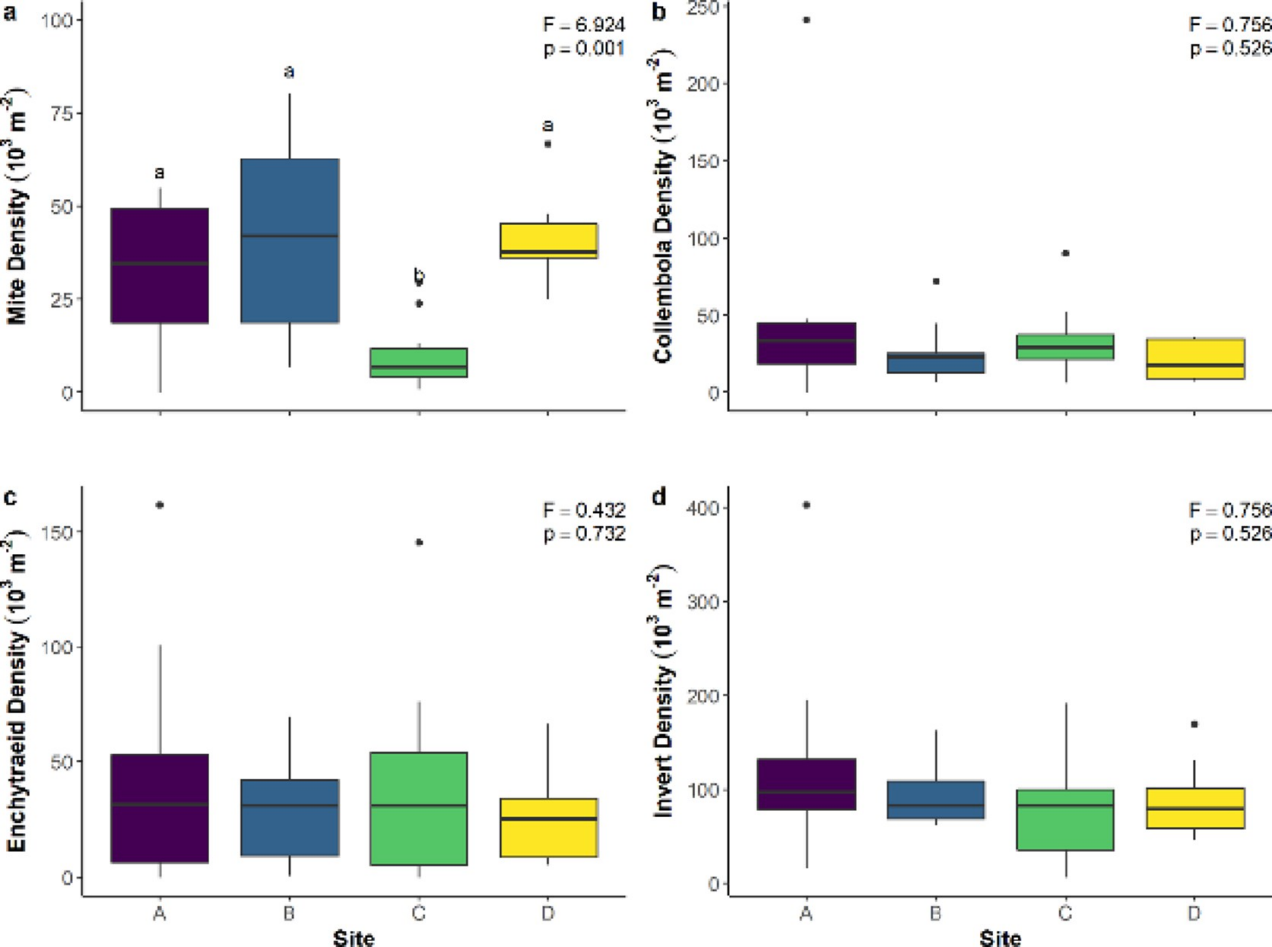
Boxplots showing the density of mites (a), collembolans (b), enchytraeids (c), and the sum of the soil invertebrates (d) in four upland tundra heath sites near Rankin Inlet, NU, Canada. Presented are the F and p values from one-way ANOVAs. Significant pairwise differences denoted by differing lowercase letters.

Invertebrate density generally varied minimally among the four sites (Fig 1 and Table 1). Indeed, collembolan, enchytraeid, and total invertebrate density did not differ among sites (Fig 1B–1D). However, mite density was significantly lower at Site C than the three other sites and tended to be less variable at Sites C and D compared to Sites A and B (Fig 1A).

### Differences in vegetation cover, substrate cover, and soil nutrients among sites

**Fig 2:**
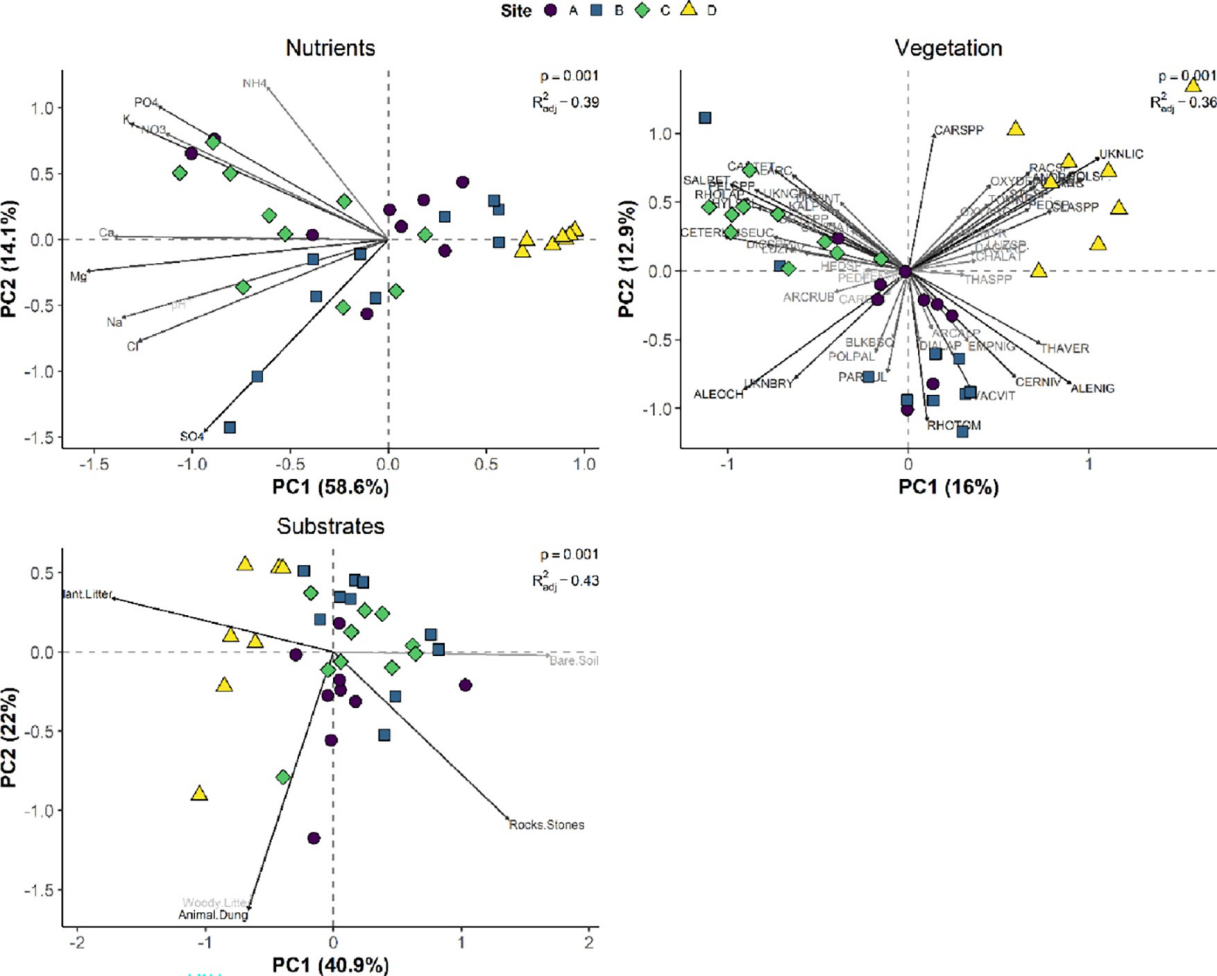
Principal components analyses of soil nutrient concentrations and pH, vegetation cover, and substrate cover among four upland tundra heath sites near Rankin Inlet, NU, Canada. The permutational multivariate analysis of variance (PERMANOVA) statistics (p-value and goodness of fit (R^2^_adj_) for differences among sites are displayed for each plot. See S1 Table for full species list and loadings for PC1 and PC2.

Vegetation cover, substrate cover, and soil nutrients (including pH) differed among the four study sites. PERMANOVA indicated that “site” had a significant (p < 0.05, R^2^_adj._ = 0.36–0.43) effect in all cases. The most evident differences occurred for vegetation cover: all sites were fairly well separated and, as such, had relatively distinct vegetation community composition (Fig 2). For soil nutrients, Site D was relatively separate from the other sites and generally had lower pH and concentrations of all nutrients, whereas Site C was, on average, was associated with the highest pH and nutrient concentrations, especially calcium (Ca^+^), phosphate (PO_4_^-^) and potassium (K^+^; Fig 2 and S2, S3 Figs). More specifically, pH was significantly lower at Site D than C and, though not significant, tended to be lower than Sites A and B. Site C had the greatest pH, though was not statistically different from Sites A and B (S3 Fig). Lastly, substrate cover was distinct among sites: Site A had greatest cover of rocks, Site B was associated with greater cover of bare soil, and Site D generally had greater cover of plant litter. Unsurprisingly based on Fig 2, the beta dispersion test for soil nutrients was significant (*F* = 5.60, *p* = 0.003); thus, we cannot disentangle the influence of differences among centroids and differences among dispersion around the centroids (e.g., beta dispersion). Beta dispersion tests for vegetation (*F* = 0.94, *p* = 0.435) and substrate cover (*F* = 2.53, *p* = 0.074) were non-significant, meaning the PERMANOVA results indicate differences among centroids of each site.

### Influence of ground cover and soil nutrients on invertebrate community

**Fig 3:**
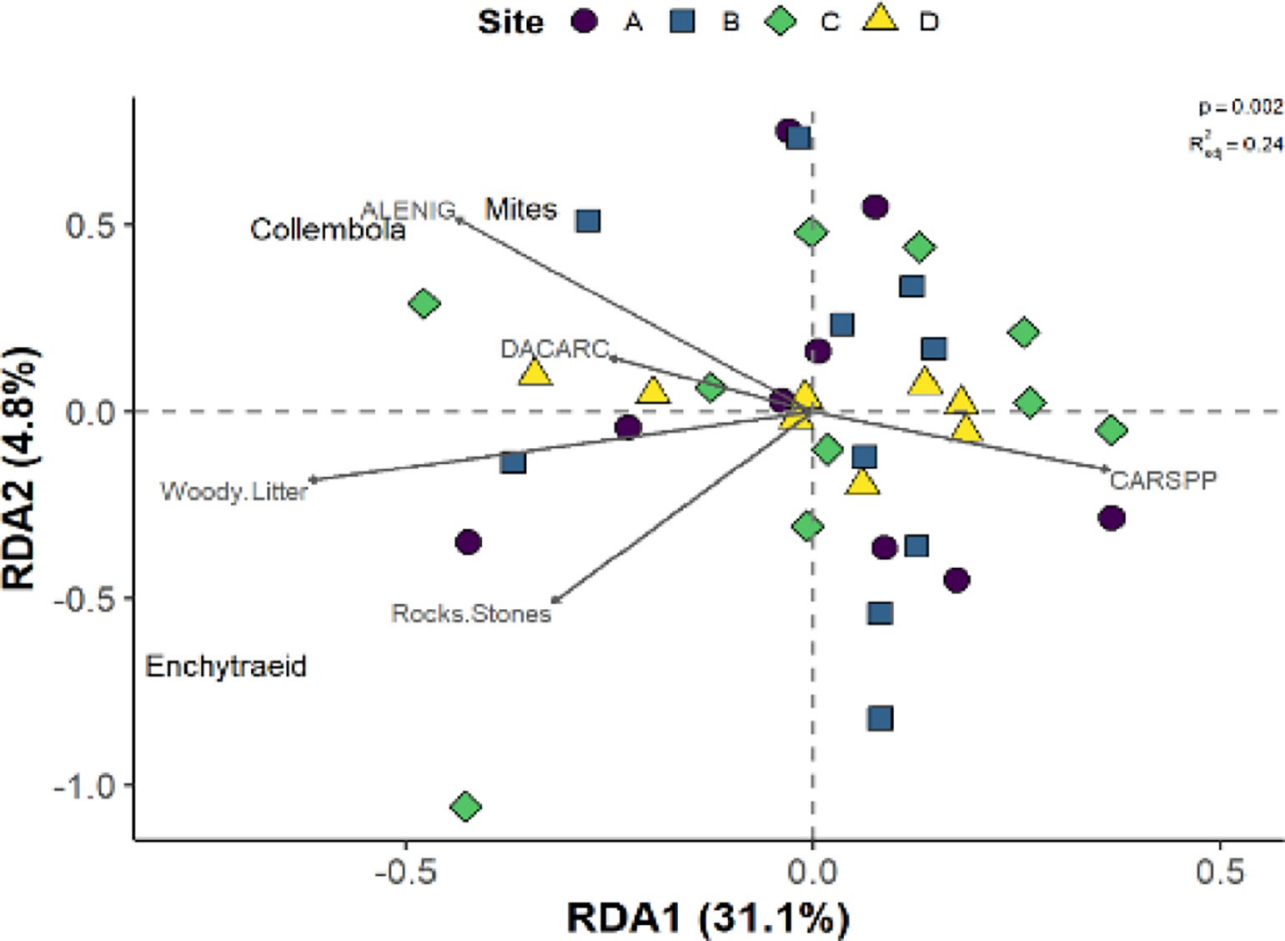
Partial redundancy analysis (RDA) demonstrating the influence of vegetation (ALENIG, DACARC, CARSPP) and substrates (woody litter and rocks) on invertebrate community density. Presented are the goodness of fit (R^2^_adj_) and p value for the RDA model. Site was used as a conditional factor. ALENIG = Alectoria nigricans (lichen), DACARC = Dactylina arctica (lichen), CARSPP = Carex spp. (graminoid).

To explore relationships among low Arctic soil invertebrates and potential environmental drivers (i.e., vegetation cover, soil nutrients and pH, and substrate cover), we conducted a partial redundancy analysis (Fig 3). Of the 24 environmental variables, five remained in the model after variable selection and three of these had significant (p < 0.05) influences on invertebrate community: cover of the lichen *Alectoria nigricans* (*F*_*1*,*28*_ = 3.81, *p* = 0.013), woody litter (*F*_*1*,*28*_ = 4.71, *p* = 0.016), rocks/stones (*F*_*1*,*28*_ = 4.13, *p* = 0.015), the graminoid *Carex* spp. (*F*_*1*,*28*_ = 2.68, *p* = 0.060), and the lichen *Dactylina arctica* (*F*_*1*,*28*_ = 1.24, *p* = 0.282). The first RDA axis was significant (*F*_*1*,*30*_ = 14.87, *p* = 0.001), as was the final model (*F*_*5*,*28*_ = 3.31, *p* < 0.001) and combined, the five variables explained about 24% of variation in invertebrate community density (R^2^_adj_ = 0.24). We found that greater cover of *A*. *nigricans*, woody litter and rocks/stones generally led to greater density of all three invertebrate species considered. Though not significant, *Carex* spp. and *D*. *Arctica* tended to have negative and positive influences on invertebrate community density, respectively. Specifically, mites and collembolans were more closely associated with cover of the two lichens, whereas enchytraeids were more closely associated with woody litter and rocks. Not surprisingly based on Fig 1 and S4 Fig, the four sites showed little separation in terms of density of the invertebrate community.

## Discussion

Overall, we found soil invertebrate communities were relatively consistent, despite clear differences in vegetation and substrate cover, and soil nutrients and pH across our tundra heath sites. However, we found that mite density was significantly lower at Site C compared to the other sites. In addition to the low mite density at Site C, this site also tended to demonstrate the highest soil pH and nutrient concentrations and a distinct vegetation community compared to the other sites. Our exploratory study also identified that cover of rocks, woody litter, and the lichen *Alectoria nigricans* had significant, positive influences on the density of all invertebrates studied. Mites and collembolans were more closely associated with cover of lichens, whereas enchytraeids were more closely associated with woody litter and rocks.

The density of invertebrates across our study was similar to that of other Arctic tundra sites [3, 8, 9, 27]. Mites averaged 10 000–42 000 individuals m^-2^ among our four upland heath tundra sites (Table 1), which closely matches average mite density across wet meadows in Alaska (10 000–83 000 m^-2^; [8] and northern Sweden (18 000 to 65 000 m^-2^; [9], mesic heath in Zackenberg, Greenland (39 000–42 000 m^-2^; [3], and across central Kola Peninsula, Russia (6200–81 160 m^-2^; [27]. Similarly, collembolan density fell within ranges reported by previous Arctic studies. We found collembolans averaged 20 500–50 000 m^-2^ among our sites, and collembolans in wet meadows of Alaska (24 000–80 000 m^-2^; [8], and in mesic heath sites of Greenland (30 700–55 000 m^-2^; [3] were similar. Enchytraeids in our study averaged 27 000–45 000 m^-2^ which is comparable to that of wet meadows in Alaska (11 000–95 000 m^-2^; [8] but far exceeds those of Sørensen *et al*. [3], who report 314–982 m^-2^.

*Alectoria nigricans* and *Dactylina arctica* are two common tundra lichens that positively influenced soil invertebrate density, especially mites and collembolans. The association of oribatid mites and lichens has long been reported [28, 29]. Several studies have found that some oribatid mite species feed on lichens and use them as shelter [30–32]. In addition, slow decomposing litter could serve as microhabitat for these organisms. Mites prefer to lay their eggs on organic matter, therefore utilizing organic matter as both a food source and preferred habitat [6, 33]. Though not significant, *Carex* spp. abundance had a negative relationship with soil invertebrates, especially mites and collembolans. We suspect this is likely a result of soil moisture, as several *Carex* species, such as *C*. *aquitilis*, tend to be more abundant in wetter conditions [34, 35]. Indeed, mites and collembolans have been shown to negatively correlate with soil moisture, whereas enchytraeids tend to be positively correlated with soil moisture [8]. Similarly, Leonov and Rakhleeva [27] reported a decrease in oribatid mite density between drier lichen tundra (23,680 m^-2^) and wetted tundra (6200 m^-2^), suggesting that wetter soil conditions are negatively influencing these soil invertebrates.

Woody litter and rock cover had positive effects on invertebrate density, especially enchytraeids. No previous studies have examined woody litter enchytraeid relationships in tundra ecosystems. However, positive associations between enchytraeid abundance and logging residues have been observed in boreal ecosystems, suggesting that enchytraeids may benefit in some way from the presence of woody litter. For example, enchytraeid abundance increased in sites where logging residues were left after clear-cutting [36–38]. Specifically, Huhta *et al*. [36] observed that old cuttings in Finnish spruce forests resulted in increased abundance of enchytraeids but no change in the abundance of micro and macro-arthropod species. Simialrly, Siira-Pietikäinen *et al*. [38] found an increase in enchytraeid density following forest harvesting, especially three-years after harvesting in clear felled sites, though collembolan density were seemingly unaffected. Although, our study site was located above the tree-line and felling did not occur in our study, the woody litter were from shrubs. Combined, these studies demonstrate an increase in enchytraeids with felled wood and support our finding of increased enchytraeid density with woody litter.

While soil nutrients and pH did not influence the invertebrate community directly, soil conditions are likely influencing the vegetation community, which in turn may influence invertebrates directly (i.e., providing litter, habitat) or indirectly by modifying soil conditions (i.e., moisture, temperature). We explored this by overlaying the nutrients as vectors (using *envfit* in *vegan*) on the vegetation PCA plot and found that most nutrients (especially nitrate, phosphate, calcium, and potassium) were positively associated with the vegetation community at Site C (S2 Fig). We also observed those same nutrients, as well as, soil pH tended to be greater at Site C (Fig 2 and S2, S3 Figs). Indeed, we found that vegetation was relatively distinct among sites (Fig 2), and Site C tended to have greater cover of *Cassiope tetragona* (evergreen shrub), *Salix reticula* and *S*. *arctica* (deciduous shrubs), *Cetraria ericetorum* (lichen), *Peltigera* spp. (lichen), and *Hylocomium splendens* (moss), as well as lower cover of *Vaccinium vitis-idaea* (evergreen shrub) and *Alectoria nigricans* (lichen) than the other three sites (S6 Fig). The differences in vegetation community at Site C may be altering litter quantity and quality, as well as modifying soil conditions. For example, *H*. *splendens* is indicative of lower light conditions and higher relative humidity at the surface [39–41], which may be influencing mite density. Though we cannot determine the precise driver(s) of lower mites at Site C, it is likely that these differences in vegetation cover are having an effect, especially because our partial RDA identified three vegetation species as important to invertebrate community, with *A*. *nigricans* having a significant, positive influence on invertebrate density. As such, the lower *A*. *nigricans* cover at Site C may be, at least in part, driving the lower mite density at this site, especially since, as previously discussed, lichen cover is known to have positive effects on mite communities [30–32]. For future research, factors such as moisture and temperature changes can be explored to see if they can explain why the factors we have identified in our study, explains invertebrate density in the tundra ecosystem.

## Conclusions

Our exploratory study revealed that soil invertebrate density is generally similar across four upland tundra heath sites in Nunavut, Canada, except for mites, which may be more strongly influenced by variation in vegetation cover than collembolans and enchytraeids. Indeed, we found that the lichen *Alectoria nigricans* had a significant, positive influence on invertebrate community and its absence may be, at least in part, driving the lower mite density found at one site. Our study provides baseline data on the density of key soil invertebrates in undisturbed upland heath and reveals potential drivers (mainly vegetation and substrate cover) of these invertebrates. Thus, with anticipated changes in vegetation occurring via anthropogenic (e.g., resource exploration and extraction) and/or natural (e.g., climate warming) disturbances, we can expect that the soil invertebrates will likewise be affected. Indeed, Bjorkman *et al*. [42] demonstrated that across 14 experimental warming studies in the Arctic, lichen abundance tended to decrease with warming. Given the positive relationship between lichen cover and invertebrate density demonstrated herein, we speculate that invertebrate density may decrease where natural and anthropogenic disturbances result in lower lichen cover. Since soil invertebrates are integral to ecosystem services like organic matter decomposition, water filtration, and carbon sequestration, these changes in invertebrate density could have far-reaching consequences for low Arctic ecosystems.

## Supporting information

S1 Data (XLSX)

S2 Data (XLSX)

S1 Table Loadings for principal component 1 and 2 of soil nutrient and pH, vegetation cover, substrate cover, and invertebrate community principal components analyses in Fig 2 and S1 Fig. * indicates variables included as predictors in RDA modelling.
(DOCX)

S1 Fig The map of the four sampling sites within the AEM Meliadine site in Nunavut, Canada. (DOCX)

S2 Fig Variation of select soil nutrients within- and among-sites at four upland tundra heath sites near Rankin Inlet, NU, Canada, demonstrating differences in key nutrients at Site C. (DOCX)

S3 Fig Variation of pH within- and among-sites at four upland tundra heath sites near Rankin Inlet, NU, Canada. Differing letters denotes significant (p < 0.05) pairwise differences between sites.
(DOCX)

S4 Fig Principal components analysis of soil inverebrate community (as density) among four upland tundra heath sites near Rankin Inlet, NU, Canada. The permutational multivariate analysis of variance (PERMANOVA) statistics (p-value and goodness of fit (R^2^_adj_) for differences among sites are displayed.
(DOCX)

S5 Fig Principal components analysis of Hellinger transformed vegetation cover data among four upland tundra heath sites near Rankin Inlet, NU, Canada (see Fig 2). Overlayed are vectors of the nutrient data from each location to assess associations among soil nutrients and vegetation cover. All vectors are significant (p < 0.05) to the analysis except for pH (p = 0.10).
(DOCX)

S6 Fig Variation of percent cover of select vegetation species within*-* and among-sites at four upland tundra heath sites near Rankin Inlet, NU, Canada, demonstrating key differences in cover at Site C. (DOCX)
