# Supplementary material for: Rocks, lichens, and woody litter influenced the soil invertebrate density in upland tundra heath
Source: PLoS One. 2023 May 2;18(5):e0282068. doi: 10.1371/journal.pone.0282068 (PMC10153722; doi:10.1371/journal.pone.0282068)
Supplement: S1 Table — * indicates variables included as predictors in RDA modelling. (DOCX) [file pone.0282068.s003.docx]

**S1 Table.** Loadings for principal component 1 and 2 of soil nutrient and pH, vegetation cover, substrate cover, and invertebrate community principal components analyses in Figure 2 and Figure S1. * indicates variables included as predictors in RDA modelling.

| **Model** | **Abbreviation** | **Variable** | **PC1 Loading** | **PC2 Loading** |
| --- | --- | --- | --- | --- |
| **Nutrients** | *pH | *Soil pH | -1.0309345 | -0.45464219 |
|  | Na | Sodium | -1.3580868 | -0.59110608 |
|  | *NH_4_ | *Ammonium | -0.6184626 | 1.15733767 |
|  | K | Potassium | -1.3159206 | 0.88067299 |
|  | Mg | Magnesium | -1.5397024 | -0.24139443 |
|  | Ca | Calcium | -1.3996978 | 0.02196742 |
|  | Cl | Chlorine | -1.2762834 | -0.77391275 |
|  | *SO_4_ | *Sulphate | -0.9399258 | -1.45808931 |
|  | *PO_4_ | *Phosphate | -1.1733842 | 1.00962061 |
|  | NO_3_ | Nitrate | -1.1345544 | 0.80321974 |
| **Vegetation** | ARCALP | *Arctostaphylos alpina* | 0.130234 | -0.41749 |
|  | *ARCRUB | **Arctostaphylos rubra* | -0.40579 | -0.15409 |
|  | CASTET | *Cassiope tetragona* | -0.73707 | 0.729488 |
|  | *DRYINT | **Dryas integrifolia* | -0.37413 | 0.49603 |
|  | EMPNIG | *Empetrum nigrum* | 0.331083 | -0.51208 |
|  | PEDFLA | *Pedicularis flammea* | -0.12879 | -0.01651 |
|  | *RHOLAP | **Rhododendron lapponicum* | -0.90515 | 0.525422 |
|  | RHOTOM | *Rhododendron tomentosum* | 0.104069 | -1.08867 |
|  | SALARC | *Salix artica* | -0.64022 | 0.697616 |
|  | SALRET | *Salix reticulata* | -0.98423 | 0.630817 |
|  | *VACMYR | **Vaccinium myrtilloides* | 0.260808 | 0.244086 |
|  | *VACVIT | **Vaccinium vitis-idaea* | 0.352368 | -0.87353 |
|  | *OXYDEF | **Oxytropis deflexa* | 0.453789 | 0.623142 |
|  | CARDIG | *Cardamine digitata* | -0.12255 | -0.17099 |
|  | LUZNIV | *Luzula nivalis* | -0.57336 | 0.131084 |
|  | PEDHIR | *Pedicularis hirsuta* | -0.0059 | -0.00144 |
|  | OXYARC | *Oxytropis arctrobia* | 0.286305 | 0.39823 |
|  | *CERNIV | **Cetraria nivalis* | 0.593337 | -0.77255 |
|  | *ALENIG | **Alectoria nigricans* | 0.899624 | -0.81814 |
|  | THAVER | *Thamnolia vermicularis* | 0.728661 | -0.52772 |
|  | POLPAL | *Polytrichum piliferum* | -0.18062 | -0.58628 |
|  | *ALEOCH | **Alectoria ochroleuca* | -0.9099 | -0.86171 |
|  | CETERI | *Cetraria ericetorum* | -1.01708 | 0.249608 |
|  | PARSUL | *Parmelia sulcata* | -0.11584 | -0.7357 |
|  | *DACARC | **Dactylina arctica* | 0.368667 | 0.136643 |
|  | *THASPP | **Thamnolia Spp.* | 0.306689 | -0.02643 |
|  | *AULTUR | **Aulacomnium turgidum* | 0.702837 | 0.614247 |
|  | BLKBSC | Black BSC | -0.08316 | -0.48409 |
|  | PELSPP | *Peltigera spp.* | -0.84639 | 0.587529 |
|  | HYLSPL | *Hylocomium splendens* | -0.83204 | 0.460976 |
|  | OXYMAY | *Oxytropis maydelliana* | -0.31219 | 0.289242 |
|  | KALPOL | *Kalmia polifolia* | -0.39977 | 0.440088 |
|  | DIALAP | *Diapensia lapponica* | 0.059559 | -0.5091 |
|  | HEDSP. | *Hedysarum sp.* | -0.23573 | 0.006692 |
|  | ANDPOL | *Andromeda polifolia* | 0.685799 | 0.649715 |
|  | PEDSP. | *Pedicularis Sp.* | 0.665682 | 0.450006 |
|  | CHALAT | *Chamaenerion latifolium* | 0.373383 | 0.075376 |
|  | RACSP. | *Racomitrium sp.* | 0.66953 | 0.716499 |
|  | TOMNIT | *Tomenthypnum nitens* | 0.443398 | 0.48593 |
|  | POLSP. | *Polytrichum sp.* | 0.884293 | 0.65778 |
|  | POAARC | *Poa Arctica* | 0.689479 | 0.607569 |
|  | STESP. | *Stellaria sp.* | 0.559915 | 0.52798 |
|  | LUZSP. | *Luzula sp.* | 0.436235 | 0.156967 |
|  | UKNGRA | Unknown grasses | -0.51419 | 0.532149 |
|  | UKNLIC | Unknown lichens | 1.056953 | 0.820037 |
|  | *CARSPP | **Carex spp.* | 0.142456 | 0.993801 |
|  | CLASPP | *Cladina spp.* | 0.7914 | 0.434006 |
|  | AUSEUC | *Astragalus eucosmus* | -0.74299 | 0.24772 |
|  | DRYSPP | *Dryas spp.* | -0.44157 | 0.346035 |
|  | *UKNBRY | *Unknown bryophytes | -0.63019 | -0.78247 |
|  | *DICSPP | **Dicranum spp.* | -0.64855 | 0.161532 |
|  | *Bare.Soil | Bare soil | 1.6900698 | -0.0222758 |
|  | *Rocks.Stones | Rocks and Stones | 1.3734685 | -1.0592055 |
| **Substrates** | *Plant.Litter | Plant litter | -1.7265111 | 0.3406602 |
|  | *Woody.Litter | Woody litter | -0.6431809 | -1.5504041 |
|  | *Animal.Dung | Animal feces | -0.6658456 | -1.6270985 |
|  | Collem | Collembolans | 368.43000 | 148.38000 |
|  | Enchy | Enchytraeids | 342.84000 | -168.74000 |
| **Invertebrates** | All_Mites | Mites | -60.81000 | 52.32000 |
