# Supplementary figures and images for: Rocks, lichens, and woody litter influenced the soil invertebrate density in upland tundra heath
Source: PLoS One. 2023 May 2;18(5):e0282068. doi: 10.1371/journal.pone.0282068 (PMC10153722; doi:10.1371/journal.pone.0282068)

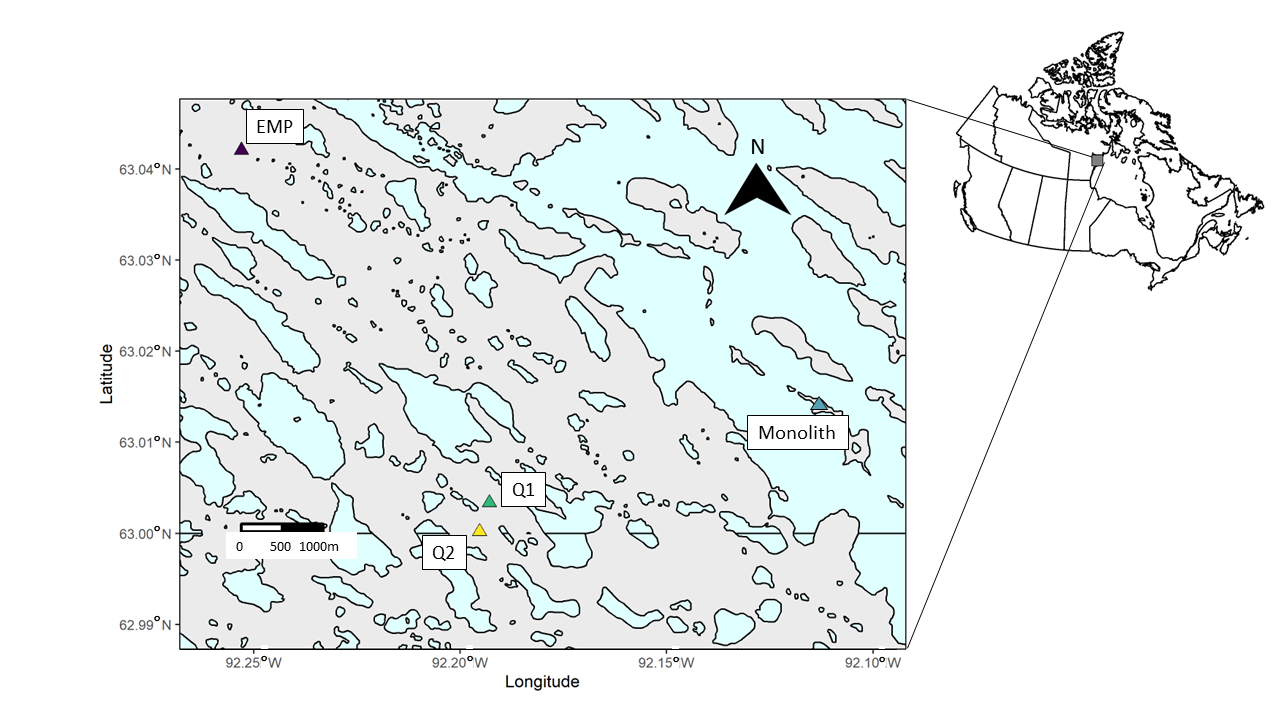


**S1 Fig.** The map of the four sampling sites within the AEM meliadine site in Nunavut, Canada.

Supplement: S1 Fig — (DOCX) [file pone.0282068.s004.docx]
