# Supplementary material for: Rocks, lichens, and woody litter influenced the soil invertebrate density in upland tundra heath
Source: PLoS One. 2023 May 2;18(5):e0282068. doi: 10.1371/journal.pone.0282068 (PMC10153722; doi:10.1371/journal.pone.0282068)
Supplement: S3 Fig — Differing letters denotes significant (p < 0.05) pairwise differences between sites. (DOCX) [file pone.0282068.s006.docx]

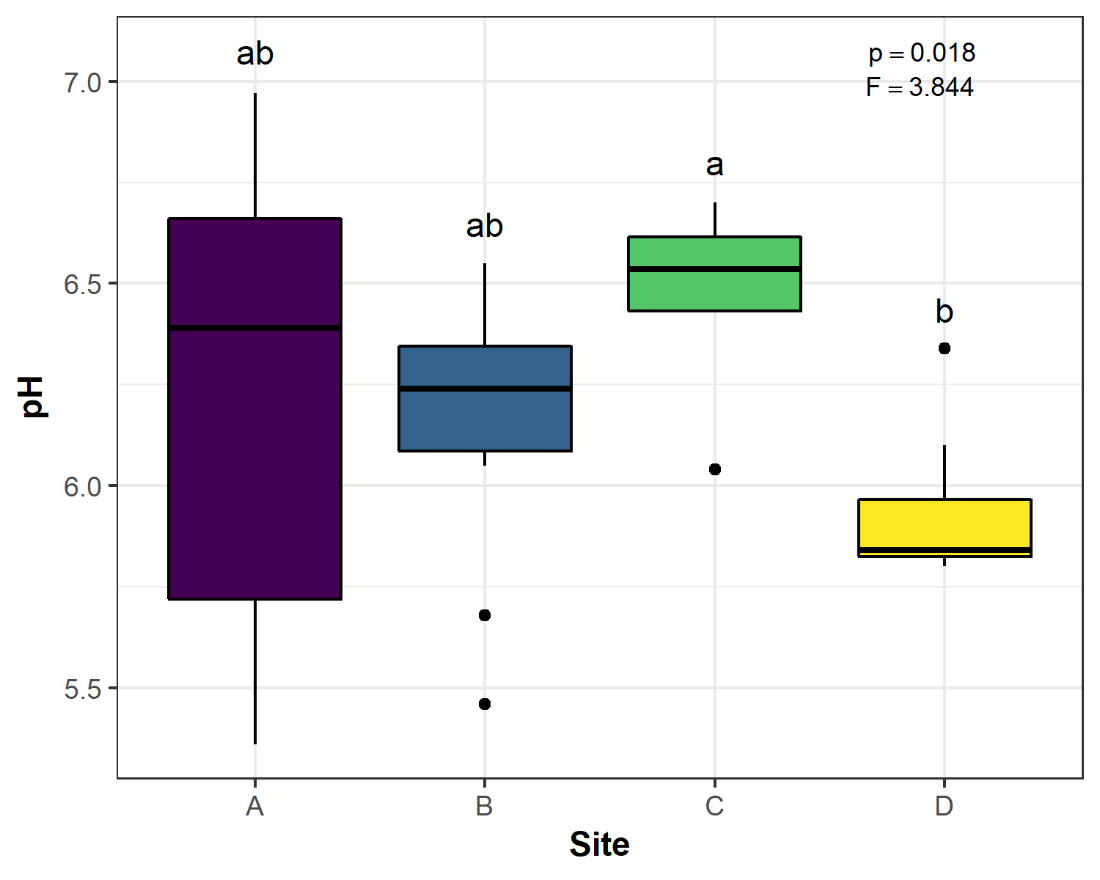


**S3 Fig.** Variation of pH within- and among-sites at four upland tundra heath sites near Rankin Inlet, NU, Canada. Differing letters denotes significant (p < 0.05) pairwise differences between sites.
