# Supplementary material for: Rocks, lichens, and woody litter influenced the soil invertebrate density in upland tundra heath
Source: PLoS One. 2023 May 2;18(5):e0282068. doi: 10.1371/journal.pone.0282068 (PMC10153722; doi:10.1371/journal.pone.0282068)
Supplement: S4 Fig — The permutational multivariate analysis of variance (PERMANOVA) statistics (p-value and goodness of fit (R2adj) for differences among sites are displayed. (DOCX) [file pone.0282068.s007.docx]

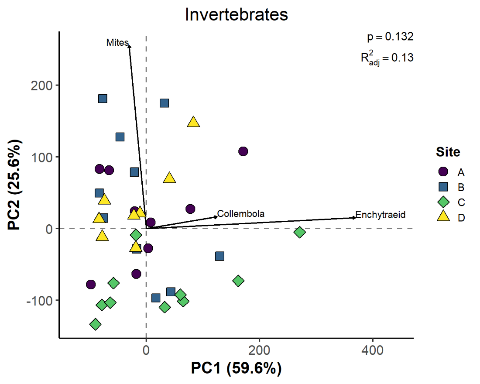


**S4 Fig.** Principal components analysis of soil inverebrate community (as density) among four upland tundra heath sites near Rankin Inlet, NU, Canada. The permutational multivariate analysis of variance (PERMANOVA) statistics (p-value and goodness of fit (R^2^_adj_) for differences among sites are displayed.
