# Supplementary material for: Rocks, lichens, and woody litter influenced the soil invertebrate density in upland tundra heath
Source: PLoS One. 2023 May 2;18(5):e0282068. doi: 10.1371/journal.pone.0282068 (PMC10153722; doi:10.1371/journal.pone.0282068)
Supplement: S5 Fig — Overlayed are vectors of the nutrient data from each location to assess associations among soil nutrients and vegetation cover. All vectors are significant (p < 0.05) to the analysis except for pH (p = 0.10). (DOCX) [file pone.0282068.s008.docx]

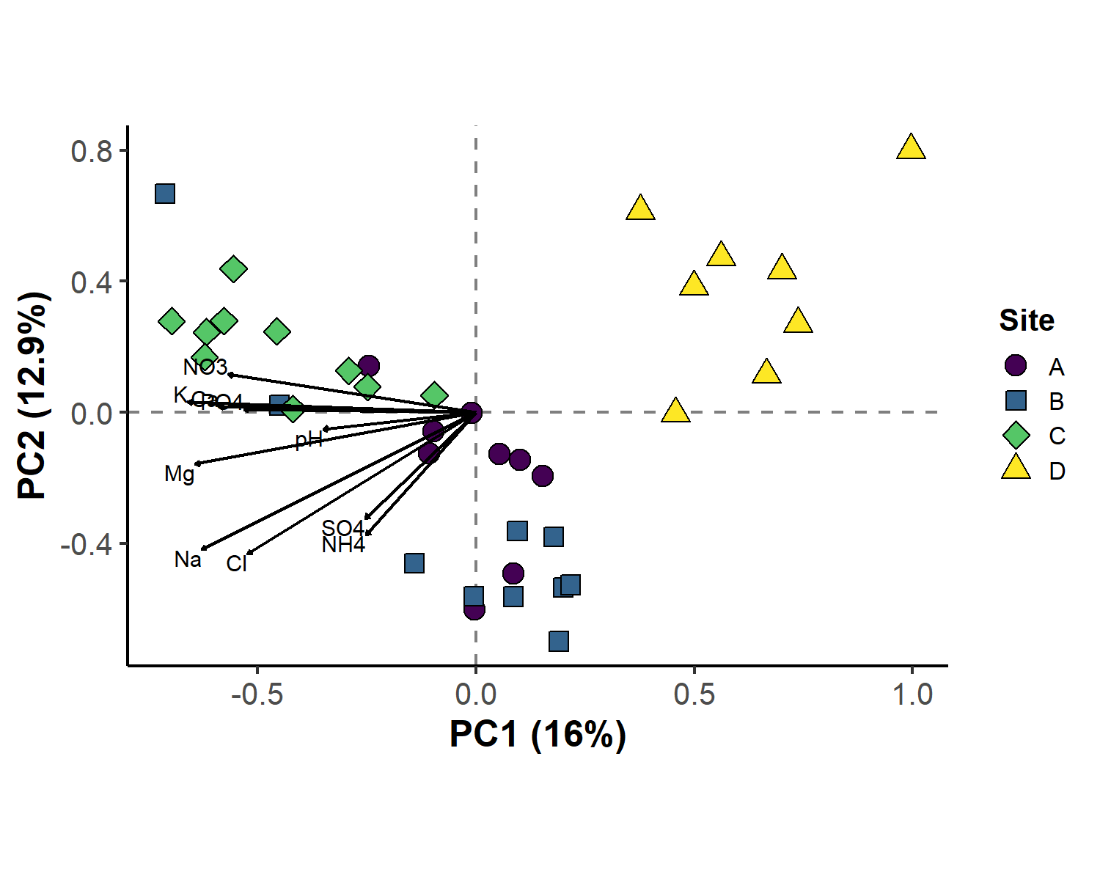


**S5 Fig.** Principal components analysis of Hellinger transformed vegetation cover data among four upland tundra heath sites near Rankin Inlet, NU, Canada (see Figure 2). Overlayed are vectors of the nutrient data from each location to assess associations among soil nutrients and vegetation cover. All vectors are significant (p < 0.05) to the analysis except for pH (p = 0.10).
