# Supplementary material for: Rocks, lichens, and woody litter influenced the soil invertebrate density in upland tundra heath
Source: PLoS One. 2023 May 2;18(5):e0282068. doi: 10.1371/journal.pone.0282068 (PMC10153722; doi:10.1371/journal.pone.0282068)
Supplement: S6 Fig — (DOCX) [file pone.0282068.s009.docx]

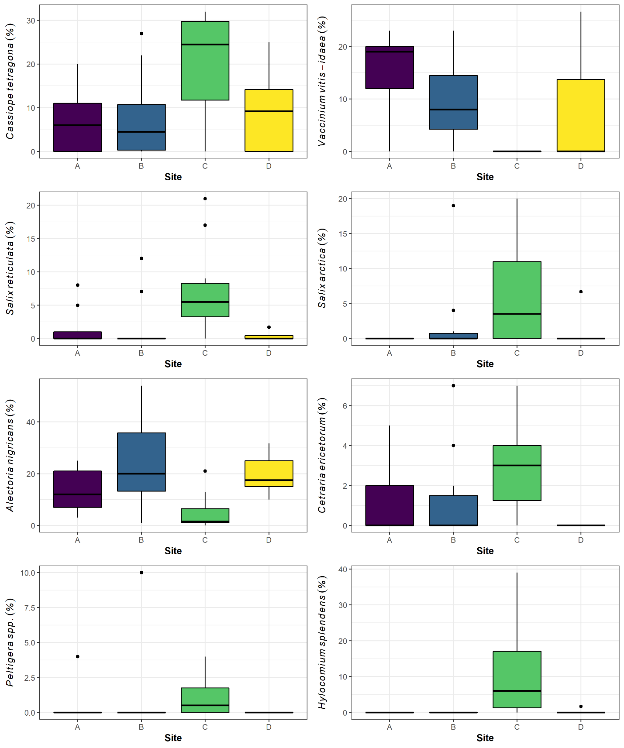


**S6 Fig.** Variation of percent cover of select vegetation species within- and among-sites at four upland tundra heath sites near Rankin Inlet, NU, Canada, demonstrating key differences in cover at Site C.
